# The B chromosome of *Sorghum purpureosericeum* reveals the first pieces of its sequence

**DOI:** 10.1093/jxb/eraa548

**Published:** 2020-11-20

**Authors:** Miroslava Karafiátová, Martina Bednářová, Mahmoud Said, Jana Čížková, Kateřina Holušová, Nicolas Blavet, Jan Bartoš

**Affiliations:** 1 Institute of Experimental Botany of the Czech Academy of Sciences, Centre of the Region Hana for Biotechnological and Agricultural Research, Šlechtitelů, Olomouc, Czech Republic; 2 University of Nottingham, UK

**Keywords:** B chromosomes, cytogenetics, flow cytometry, pollen nuclei, repeat analysis, *Sorghum purpureosericeum*

## Abstract

More than a century has passed since the B chromosomes were first discovered. Today we know much of their variability, morphology, and transmission to plant progeny. With the advent of modern technologies, B chromosome research has accelerated, and some of their persistent mysteries have since been uncovered. Building on this momentum, here we extend current knowledge of B chromosomes in *Sorghum purpureosericeum* to the sequence level. To do this, we estimated the B chromosome size at 421 Mb, sequenced DNA from flow-sorted haploid pollen nuclei of both B-positive (B+) and B-negative (B0) plants, and performed a repeat analysis on the Illumina raw sequence data. This analysis revealed nine putative B-specific clusters, which were then used to develop B chromosome-specific markers. Additionally, cluster SpuCL4 was identified and verified to be a centromeric repeat. We also uncovered two repetitive clusters (SpuCL168 and SpuCL115), which hybridized exclusively on the B chromosome under fluorescence *in situ* hybridization and can be considered as robust cytogenetic markers. Given that B chromosomes in *Sorghum* are rather unstable across all tissues, our findings could facilitate expedient identification of B+ plants and enable a wide range of studies to track this chromosome type *in situ*.

## Teaser

We sequenced plants of the tropical grass *Sorghum purpureosericeum* carrying B chromosomes. Based on repeat clustering, we assigned nine clusters to B chromosome and developed B-specific PCR and cytogenetic markers.

## Introduction

B chromosomes are special genomic components with a fate of their own. They are dispensable parts of the genomes that escape Mendelian inheritance and mostly bring no advantage to the host but show strong negative effects on it when present in higher numbers. Nevertheless, there are a few species with fitness advantage of having a B chromosome reported, namely chives *Allium schoenoprasum* (Plowman and Bougourd, 1994) or fungal pathogens *Nectria haematococca* (Han *et al.*, 2001) and *Magnaporthe oryzae* (Peng *et al.*, 2019). The selfish nature of these supernumerary chromosomes secures their persistence in the population via the mechanism of non-disjunction, which goes directly against natural selection (Jones *et al.*, 2008). Much is now known about B chromosomes in general, yet we still have only vague knowledge of their significance to their carriers as well as of their emergence and evolution.

Despite the topic of supernumerary chromosomes becoming more conspicuous over the last three decades, its research scope across species remains largely uneven. Most of the assessed information still comes from a few favoured species of certain economic importance, making them preferred research targets. Being neither a staple food nor contributing extra nutrients in the human diet, wild sorghum plants have lingered on the periphery of science thus far and so our knowledge of these species is very poor. Nevertheless, knowing more about these wild sorghums may be beneficial given the rising demand for cultivated *Sorghum bicolor* in developing countries, particularly in tropical Africa, where *S. bicolor* is among the staples for rural people (Taylor, 2004; FAO, 1995). Wild sorghums represent a valuable tertiary genetic pool that is useful for breeding. Notably, perennial species are favoured by breeders aiming to produce and introduce perennial *S. bicolor* cultivars (Piper and Kulakow, 1994; Dweikat, 2005).

Though the exact number of species with reported supernumeraries is unknown (D’Ambrosio *et al.*, 2017), the total number is estimated at >2800 species widespread in all kingdoms. Of these, the vast majority have been reported from plants (Ahmad and Martins, 2019), with maize (Kuwada, 1925; Longley, 1927; Kato *et al.*, 2005; Carlson, 2007; Su *et al.*, 2018) and rye (Gotoh, 1924; Martis *et al.*, 2012; Banaei-Moghaddam *et al.*, 2019) receiving the most research attention.

The B chromosomes in the genus *Sorghum* remain almost entirely unexplored. Nearly all published reports focus exclusively on elementary karyological descriptions and B chromosome transmission via meiosis. Among the 22 *Sorghum* species, B chromosomes were reported in just five. First, they were discovered in *S. verticilliflorum* in 1934 (Huskins and Smith 1934) and later in *S. purpureosericeum* (Janaki-Ammal, 1939), *S. nitidum* (Raman and Krishnaswami, 1959), *S. halepense* (Raman *et al.*, 1964), and most recently in *S. stipoideum* (Wu, 1992). Apart from *S. verticilliflorum*, whose B chromosome was described as a fragment, the species’ B chromosomes are of a standard size or are shorter when compared with chromosomes from the A complement. A maximum of six B chromosomes tolerated by the cell was recorded in *S. purpureosericeum* and *S. halepense* (Janaki-Ammal, 1940; Raman *et al.*, 1964).

Progress in acquiring more knowledge of accessory chromosomes in *Sorghum* spp. is challenging because of their high numerical instability. Generally, the transmission of B chromosomes during plant development and growth is regular in somatic cells. Nevertheless, there are exceptions to this rule. In *Crepis capillaris*, the numbers of B chromosomes in aerial parts differs from those in roots and claudicle leaves (Rutishauser and Röthlisberger, 1966). Somatic variation in B chromosome frequency was also found in both *Poa alpina* and *Agropyron cristatum*, for which B chromosomes are absent in adventitious roots yet preserved in primary roots (Müntzing and Nygren, 1955; Baenzinger 1962). Further, the elimination of B chromosomes from all roots is a specific phenomenon described in few plant species, namely *Aegilops* (Mochizuki, 1957; Mendelson and Zohary, 1972), *Haplopapus gracilis* (Östergen and Fröst, 1962), or *Poa timoleontis* (Nygren, 1957). Similarly, in *S. purpureosericeum*, the B chromosomes do not occur in roots, and are unstable in young shoots, ovaries, and tapetal cells. Their fixed distribution has been demonstrated only in fertile florets (Janaki-Ammal, 1940; Darlington *et al.*, 1941). Recently, the mechanism of tissue-specific B chromosome distribution was uncovered in *Aegilops speltoides* (Ruban *et al.*, 2020). This study reveals that B chromosome elimination from roots in goatgrass is a programmed and controlled process with onset only few days after pollination. It shows that elimination of the B chromosome is a consequence of non-disjunction of B chromatids in anaphase (Ruban *et al.*, 2020). Interestingly, similar numerical variability has never been observed either in rye or maize, whose distribution of B chromosomes is stable during their life cycle and the B chromosomes can be found in all common tissues (Jones and Rees, 1982).

Here, we report on the first attempt to gain a deeper insight into the genome of *Sorghum purpureosericeum* at the sequence level. Our study estimated B chromosome size and discovered a scattered shotgun sequence, which was acquired to derive B-specific markers. This is an essential prerequisite for broader studies to elucidate the mechanism(s) underpinning the biology and evolution of accessory chromosomes in wild sorghums.

## Materials and methods

### Plant material

The seeds of *S. purpureosericeum* (Hochst. ex A.Rich.) Schweinf. & Asch. (accession no. IS18947) with an unknown B chromosome status were supplied by the International Crop Research Institute for the Semi-Arid Tropics (ICRISAT, India). Before sowing them, the hard seed coats were removed and seeds were soaked in water overnight. Seeds were germinated on Petri dishes in a thermal incubator under a 8 h light/16 h dark photoperiod at temperatures of 29 °C day/25 °C night. The ensuing seedlings were planted into soil mixed with sand (2:1) in 10 cm diameter pots and cultivated under the same conditions as for seeds.

### Population screening

To distinguish those plants carrying B chromosomes, haploid nuclei from their pollen grains were isolated and analysed using flow cytometry. Briefly, a few fresh anthers from individual plants were collected and their pollen extracted in a LB01 buffer (Doležel *et al.*, 1989) by vortexing for 5 min at 10 000 rpm. Additionally, samples were shaken for another 12 min in a thermomixer (Eppendorf, Hamburg, Germany) at 2000 rpm and 25 °C to release any residual pollen grains from the anthers. Pollen grains were separated from open anthers by centrifugation at 850 *g* for 5 min, at 20 °C. The empty anthers were removed with tweezers, and pollen nuclei were released from tough pollen by adding glass beads (Sigma Aldrich, Cat. No. G8772) and vortexing this for 5 min at 10 000 rpm. The resulting suspension containing the haploid nuclei was then filtered through 20 µm mesh, stained with DAPI (2 μg ml^–1^), and analysed using FACSAria SORP (BD Biosciences, San Jose, CA, USA).

The presence of the B chromosomes in plants analysed using flow cytometry was verified in meiosis for 10 B-negative (B0) individuals and all B-positive (B+) individuals. Immature anthers were collected and checked for their developmental stage. Anthers at meiotic metaphase I were fixed in 3:1 (ethanol:glacial acetic acid) for 7 d at 37 °C, and then stored in 70% ethanol at –20 °C. The number of bivalents was determined on simple squashed preparations (Masoudi-Nejad *et al.*, 2002) stained with DAPI under a fluorescent microscope Zeiss Axio Image Z2.0. For each plant, 50 meiocytes in metaphase I were scored to analyse the stability and number of B chromosomes present.

### B chromosome size estimation

Nuclear genome size was measured using flow cytometry according to Doležel *et al*. (2007). With respect to B chromosome size estimation, we first estimated the genome size of B0 plants of *S. purpureosericeum* (IS18947). Samples for genome size estimation were prepared from young leaves and analysed three times on three different days. *Zea mays* cv. CE-777 with an estimated genome size of 5.43 pg/2C served as an internal reference standard. Genome size (2C value) was determined considering that 1 pg of DNA is equal to 0.978×10^9^ bp (Doležel *et al.*, 2003).

Similarly, nuclei for estimation of B chromosome size were isolated from florets of 1B plants. The genome size of 1B plants was calculated from the ratio of peak positions for the two populations of nuclei (2C versus 2C+1B) based on the estimate of the genome size of B0 plants of *S. purpureosericeum* (IS18947). B chromosome size was determined by subtracting the genome size of *S. purpureosericeum* B0 plants from the genome size of 1B plants.

### Isolation and sequencing of pollen nuclei

Haploid pollen nuclei from both B+ and B0 plants (accession no. IS18947) were isolated using flow cytometry. The samples were prepared and processed as described above. From each individual, 9000 of its 1C nuclei (corresponding to 20 ng of DNA) were flow-sorted into 40 µl of distilled water in a 0.5 ml tube; nuclear DNA was used separately for preparing the sequencing libraries. Sorted nuclei were treated with 1.8 µl of proteinase K (10 mg ml^–1^), for 18 h at 50 °C, to perform the protein degradation. Proteinase K was deactivated at 85 °C for 15 min, after which the samples were frozen at –80 °C. After adding water up to 100 µl, DNA from nuclei was fragmented using a Bioruptor Plus (Diagenode, Denville, NJ, USA); this was done six times, for 30 s each, at its high setting. Fragmented DNA was purified with Ampure XP (Beckman Coulter, Brea, CA, USA). Libraries for sequencing were prepared using the NEBNext® Ultra™ II DNA Library Prep Kit for Illumina (Ipswich, MA, USA) with the following modifications: (i) size selection was directed to obtain a larger final library size (~1000 bp) and (ii) PCR enrichment was carried out in 12 cycles. Finally, the prepared libraries were size selected within a range of 700–1000 bp using a Blue Pippin Instrument (Sage Science, Beverly, MA, USA). Libraries were sequenced on NovaSeq 6000, and 2×250 bp paired-end reads were eventually produced.

### Repeat analysis

Analysis of repetitive sequences was implemented using the Galaxy-based server (Afgan *et al.*, 2018), supplemented with the RepeatExplorer2 (Novák *et al.*, 2013) and TAREAN (Novák *et al.*, 2017) tools (https://repeatexplorer-elixir.cerit-sc.cz). Sequence reads were pre-processed in this way: reads whose quality was not greater than 10 for >95% of bases were removed, as were any reads having ambiguous nucleotides (Ns); then, the first 20 nucleotides were removed and the reads were trimmed to the same length of 150 bp. Sequence data were down-sampled to 600 000 paired-end reads (2×150 bp) from each sample-B0 and B+. This corresponds to the equivalent of ~8% of the genome, assuming a genome size of 2.21 Gb/1C. Next, the reads of both samples were clustered with RepeatExplorer2, and putative tandem repeats were analysed with TAREAN. Clusters representing >0.01% of the entire dataset were investigated further, manually. Sequence reads used in this analysis, the resulting sequences of clusters, and the counts of B+/B0 reads in each cluster have all been submitted to the Dryad public repository (https://doi.org/10.5061/dryad.rxwdbrv5j; Karafiátová *et al.*, 2021).

### PCR marker development

Nine putative B-specific clusters were revealed by the repeat analysis (Table 1). For each cluster, specific primers with short amplicons (up to 1.3 kbp, Supplementary Table S1) and long amplicons (in the range of 2–5 kbp, Supplementary Table S2) were designed using Primer3 software (Koressaar *et al.*, 2007; Untergasser *et al.*, 2012). Long amplicons were found to be missing for clusters SpuCL135, SpuCL144, CL 168, and SpuCL214 due to the limited length of their cluster sequence.

**Table 1.** Characteristics of B-specific/enriched repetitive clusters and centromeric satellites as revealed by the RepeatExplorer2/TAREN pipeline

Cluster	Consensus length (bp)	Genome proportion	Annotation	B-specific reads	PCR specificity	FISH signal
SpuCL4	137	1.00%	Centromeric repeat	58.6%	B0/B+	Centromeres
SpuCL115	N/A	0.20%	Class_II./hAT	99.4%	B+	B specific
SpuCL135	1 130	0.14%	Putative satellite	100%	B0/B+	
SpuCL144	366	0.11%	Putative satellite	99.9%	B0/B+	
SpuCL168	1 560	0.056%	Putative satellite	100%	B+	B specific
SpuCL169	6 860	0.055%	Putative satellite	98.2%	B+	B/As
SpuCL175	N/A	0.048%	Class_I./LINE	97.8%	B+	B/As
SpuCL189	3 500	0.028%	Putative satellite	94.7%	B+	B/As
SpuCL214	260	0.017%	Putative satellite	100%	B+	No signal
SpuCL220	N/A	0.015%	N/A	89.9%	No product	

Primer specificity was tested on B+ genomic DNA, for which B0 genomic DNA served as the control. DNA was extracted from whole, lyophilized spikelets of *S. purpureosericeum* plants (IS18947), by using the NucleoSpin® Plant II Kit (Macherey-Nagel, Düren, Germany) and following the manufacturer’s recommendations. DNA concentration was measured using a Nanodrop® ND-1000 spectrophotometer (Saveen Werner, Malmo, Sweden).

Long amplicons were amplified with PrimeStar®GXL DNA polymerase (Takara Bio Inc., Shiga, Japan) on a C1000 Touch™ Thermal Cycler (BioRad, Hercules, CA, USA). Each PCR (20 μl) contained 50 ng of genomic DNA, 1× PrimeSTAR GXL buffer, 0.2 mM dNTPs, 0.25 μM of each primer, 0.5 U of PrimeSTAR GXL DNA polymerase, and distilled water. The products were amplified in 30 cycles, consisting of 98 °C/10 s, 60 °C/15 s, and 68 °C/270 s, and separated on a 0.8% agarose gel.

Short amplicons were amplified using *Taq* DNA polymerase with the Standard Taq Buffer (New England Biolabs, Ipswich, MA, USA). Each PCR contained 20 ng of genomic DNA, 1× Standard Taq Reaction Buffer, 0.2 mM dNTPs, 0.5 μM of each primer, 0.5 U of *Taq* DNA polymerase, and distilled water up to 20 μl. The reaction was conducted under these conditions: initial denaturation 98 °C/3 min; 35 cycles of 98 °C/30 s; 60 °C (62 °C)/30 s; 72 °C/60 s, followed by a final extension at 72 °C/5 min. The annealing temperature (*T*_a_) was determined to be 60 °C for all primers, except for SpuCL144 and SpuCL214 (*T*_a_=62 °C). The amplification products were visualized on a 1.5% agarose gel.

### Microscopic slide preparation, probe labelling, and fluorescence *in situ* hybridization (FISH)

Meiotic chromosome spreads for FISH were prepared from fixed immature anthers of B+ plants and B0 plants serving as negative control. The anthers were first digested with 4% cellulase Onozuka R-10 (Yakult Honsa Co. Ltd, Minato-ku, Japan) and 1% Pectolyase Y-23 (MP Biochemicals, Santa Ana, CA, USA,) in a 1× KCl (0.15 M KCl, 5.4 mM EDTA, pH 4) buffer for 20 min at 37 °C. Macerated anthers were squashed and the slides prepared as described by Karafiátová *et al.* (2013).

All six clusters resulting in a B-specific product were tested as cytogenetic markers. Where possible, long amplicons were preferably used for probe preparation. For clusters SpuCL115, SpuCL169, SpuCL175, and SpuCL189, the long PCR products were labelled with aminoallyl-dUTP-5-FAM (fluorescein aminide; Jena Biosciences, Jena, Germany) or Texas Red d-UTP (Invitrogen, Camarillo, CA, USA), using the Nick translation Mix (Roche, Mannheim, Germany). In clusters SpuCL214 and SpuCL168, where no long amplicons could be designed, the probes were labelled using PCR. Additionally, cluster SpuCL4 was identified as a centromeric repeat. The centromeric probe and B-specific clusters SpuCL168 and SpuCL214 were labelled with fluorescein isothiocyanate (FITC)–dUTP (Roche, Mannheim, Germany) or Texas Red-dUTP (Invitrogen), using PCR. The 25 µl reaction consisted of 30 ng of genomic DNA, 1 µM of each forward and reverse primer (primer sequences for SpuCL214 and SpuCL168 are listed in Supplementary Table S1; primers for centromeric cluster SpuCL4 were as follows: F, AGTGGAAGCACGTTTCGGTA; R, ATCGGGTGCATCCAAAACTA), 1× *Taq* polymerase buffer, nucleotides (0.2 mM each of dATP, dCTP, dGTP; 0.1 mM each of dTTP and dUTP), and 0.5 U of *Taq* polymerase (New England Biolabs). The labelling was implemented under these conditions: initial denaturation, at 94 °C/60 s; 30 cycles of 94 °C/30 s, 60 °C/30 s, 72 °C/30 s; final extension, at 72 °C/10 min.

The slides with mounted chromosome preparations were fixed in 4% formaldehyde, as described in Said *et al.* (2018). Hybridization was performed following Karafiátová *et al.* (2013) and the signals were evaluated using Zeiss Axio Image Z2.0 equipped with a Cool Cube camera (Metasystems, Altussheim, Germany) and an appropriate set of filters.

## Results and discussion

### B chromosomes are consistently detectable in the ‘germ line’ only

**Fig. 1.**
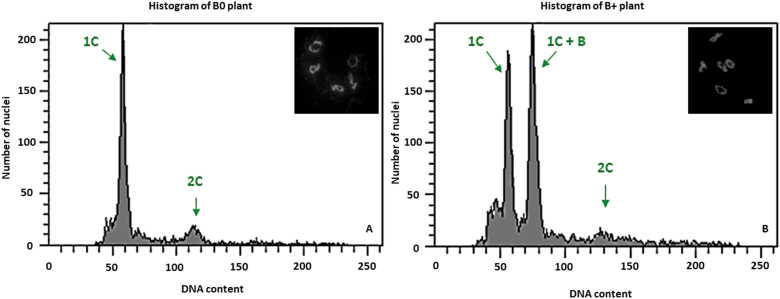
Flow histograms of pollen nuclei isolated from B0 (A) and 2B (B) plants of *Sorghum purpureosericeum*. Insets show the number of bivalents in meiotic metaphase I of analysed plants. (A) Histogram of a B0 plant with two peaks representing haploid 1C pollen nuclei and 2C nuclei corresponding to residual cells from tapetum. (B) The population of haploid cells splits into two distinct subpopulations representing those harbouring 1C and 1C+B nuclei.

Because the seed collections available in genebanks lack information about B chromosome status, here we screened anonymous accessions of *S. purpureosericeum*, with a preference for those originating in Sudan and India where the B chromosome(s) were previously reported (Janaki Ammal, 1939; Wu, 1984). Following the finding from old reports that B chromosomes were absent in roots, shoots, and leaves (Darlington *et al.*, 1941), we analysed the meiocytes and pollen nuclei in anthers; however, the cytological scoring of meiocytes is rather laborious and time consuming. To circumvent this limitation, using flow cytometry, we instead checked parts of whole plants for the presence of B chromosomes. Detection of B chromosomes in plant tissues using flow cytometry has been previously employed in goatgrass, where this approach turned out to be reliable to detect B chromosomes in leaves (Wu *et al.*, 2019) and even in immature embryos (Ruban *et al.*, 2020). In our material, the population of B+ nuclei formed a distinct, clearly resolved peak in samples prepared from anthers (Fig. 1) and the peduncle (Supplementary Fig. S1). Further, we found a residual population of B+ nuclei also in leaf meristems and the last node. Other tissues such as root, leaf, and stem were found to be lacking B chromosomes (Supplementary Fig. S1). Considering the results of our sampling, detection of the presence of B chromosomes relies on the proportion of B+/B0 nuclei in individual parts/tissues and thus on developmental stage. The results indicate that B chromosomes could be partially preserved in meristems. However, the amount of B+ nuclei is frequently below the detection limit of the approach. As such, we can state that B chromosomes are consistently detected in the cell line leading to the reproductive organs. Taking all recent and older findings into consideration, the hypothesis of B chromosome elimination accompanying cell differentiation could be reasonably proposed. This would be the first example of such B chromosome behaviour in plants. Nevertheless, the B chromosome elimination from somatic cells in adults was described earlier for the flatworm *Polycelis tenuis* (Melander, 1950). In a myricine ant, *Leptothorax spinosior*, the eradication of B chromosomes has even been extended and their distribution shown to be stable only in male germ lines (Imai, 1974).

**Fig. 2.**
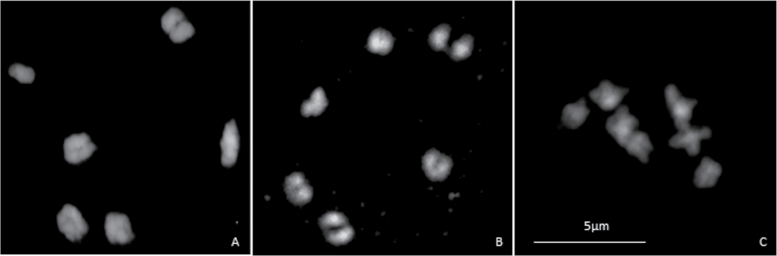
Meiosis in B+ plants of *Sorghum purpureosericeum*. (A) B+ plant (2*n*=2*x*=10+B) forming five bivalents and one univalent in meiotic metaphase I; (B) B+ plant (2*n*=2*x*=10 + 2B) showing six ring bivalents; and (C) B+ plant (2*n*=2*x*=10 + 4B) with seven bivalents.

In our study, a total of 120 plants were checked for their B chromosome status using flow cytometry. Of them, 19 individuals (16%) showed distinct additional peaks in the histogram, whereas in plants lacking the B chromosome in anthers two peaks appeared, representing haploid 1C pollen nuclei and 2C nuclei corresponding to residual cells from the tapetum (Fig. 1A). Specifically, the B+ individual population of haploid cells had split into two distinct subpopulations representing 1C and 1C+B nuclei (Fig. 1B). Occasionally, a population of 2C nuclei was also observed, most probably being nuclei from anther walls, where the B chromosome distribution is variable. The status of plants was further evaluated cytogenetically in metaphase I. In analysed plants showing no extra population in the histogram, five bivalents were observed in all meiocytes, confirming the absence of B chromosomes. In contrast, plants showing extra populations always had more than five bivalents. The B chromosomes formed univalents (if odd in number, Fig. 2A) or ring bivalents (Fig. 2B). Notably, rod bivalent formation was observed. A maximum of four B chromosomes were found in our plant material (Fig. 2C) Further, the B chromosome was present in all meiocytes at metaphase I in B+ individuals, and the number of B chromosomes in all analysed cells was stable.

In addition to its numerical traits, structural polymorphism is a characteristic phenomenon of B chromosomes. More than one polymorphic form has been reported in >60 species (Jones and Rees, 1982). Although a polymorphism in size had been observed before in *S. purpureosericeum*, we have never seen such variability. All B chromosomes observed in our material were of the same size, being on a par with standard A chromosomes. Interestingly, the reports describing B polymorphism in *S. purpureosericeum* are not in agreement. Although Reddy *et al.* (1958) found two B chromosome types in his material: a short B chromosome (S) similar in size to A and an isochromosome (L) twice as large, Darlington *et al.* (1941) unveiled more additional types that probably arose from a morbid mitosis. Besides the sorghums, two polymorphic types of B chromosomes, metacentric and telocentric, were detected for instance in *Aegilops mutica* (Mochizuki, 1960). Further, there are six forms of Bs in rye (Jones and Rees, 1982), and up to 29 forms occur in *A. schoenoprasum* (Bougourd and Parker, 1979).

### Accumulation of specific repetitive sequences in the B chromosomes of *S. purpureosericeum*

**Fig. 3.**
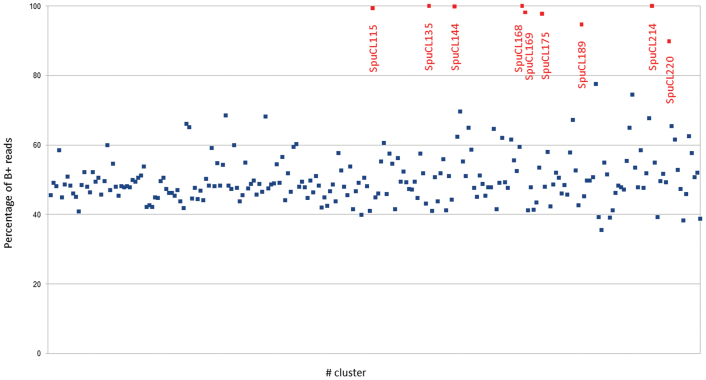
Proportion of reads from the B+ sample in clusters generated and analysed using the RepeatExplorer pipeline. The clusters are organized along the *x*-axis based on their frequency in the genome (i.e. leftmost being most frequent). Values on the *y*-axis correspond to percentage of reads from the B+ sample. Clusters labelled in red are expected to be specific/enriched in the B+ sample. Note that the value should be ~50% for sequences (clusters) that originated in the A chromosome complement.

Isolated haploid nuclei of B+ and B0 plants were sequenced in this study. Our analysis of repetitive sequences was done using the RepeatExplorer2 pipeline (Novák *et al.* 2013). Out of 2.4 million reads, a total of 1 952 433 were analysed (975 698 from the B+ sample and 976 734 from the B0 sample). Among them, 66% were clustered, leaving 432 108 reads occurring as singlets. The analysis generated 231 clusters, each accounting for at least 0.01% of the original dataset. Comparing the count of reads in these clusters (between B+ and B0 samples) revealed that individual clusters consisted of 35.6–100% reads of the B+ sample. Among them, 194 clusters had a nearly equal representation (40–60%) of reads from the B+ sample as from the B0 sample, a ratio expected for clusters originating in the A chromosome complement (as it is present in both samples). In contrast, just nine clusters were significantly enriched for reads of the B+ sample, whose proportion was at least 80% (Fig. 3; Table 1). Sequences of these clusters were further checked for B specificity, with subsequent annotations of cluster sequences based on a protein domain search within the pipeline. This revealed one B-specific DNA transposon/hAT (SpuCL115) and one long interspersed nuclear element (LINE; SpuCL175). Putative tandem repeats (satellites) were identified based on cluster layout topology, using the TAREAN tool (Novák *et al.*, 2017). For those clusters, k-mer frequencies in a set of oriented reads obtained from the graph were used to reconstruct the consensus sequence of the satellite monomer. Among putative B-enriched sequences, six qualified as being low-confidence satellites (SpuCL135, SpuCL144, SpuCL168, SpuCL169, SpuCL189, and SpuCL214; Supplementary Fig. S2). Similarly, B-specific repeats have been identified in the sequence of other plant B chromosomes (Alfenito and Bichler 1993; Martis *et al.*, 2012; Wu *et al.*, 2019; Ebrahimzadegan *et al.*, 2019). Moreover, our analysis of repetitive sequences revealed that one of the most abundant clusters (SpuCL4) is a promising candidate sequence for a centromeric satellite repeat.

### B chromosome sequence reveals its specific landmarks

**Fig. 4.**
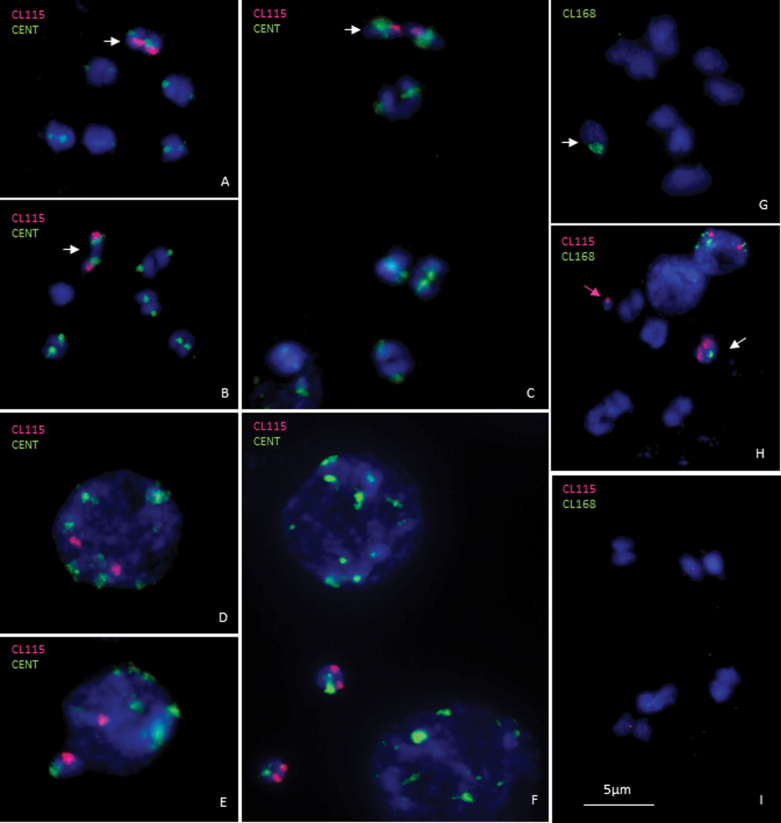
FISH with the B-specific probes SpuCL115 and SpuCL168 and the centromeric probe SpuCL4. The SpuCL115 (red) and SpuCL4 (green) on 2B plants with B chromosomes forming ring bivalents (A) and rod bivalents with an opposite orientation (B, C) in meiotic metaphase I. Two distinct SpuCL115 (red) signals and a centromere (green) on the interphase nucleus (D). The SpuCL115 (red) on the nucleus with one B chromosome preserved and on the micronucleus with one B chromosomes eliminated (E); SpuCL115 signals on micronuclei with both B chromosomes, for which adjacent nuclei are free from signals (F). The B-specific cluster SpuCL168 (green) in meiotic metaphase I (G); co-localization of SpuCL115 (red) and SpuCL168 (green), with the purple arrow pointing to the micronucleus (H). Localization of B-specific probes on meiotic metaphase I of B0 plants showing no distinct signal (I). Chromosomes are counterstained with DAPI (blue) and B chromosome bivalents are indicated by arrows.

In some species, specific morphological features will let researchers distinguish the B chromosome in the karyotype based on simple cytological observations. Often, it is their size that is distinct, and this conspicuous trait can be gleaned in species with smaller B chromosomes, as in *Secale cereale* (Jones, 1991) and *A. cristatum* (Said *et al.*, 2019), or, conversely, those with B chromosomes larger than A chromosomes, such as in *Plantago serraria* (Frost, 1959) and *Rumex thyrsiflorum* (Zuk, 1969). Occasionally, the position of the centromere could serve as a landmark. The supernumeraries were described as telocentrics in *Allium pulchellum* (Tschermak-Woess and Schinmann, 1960) or *P. alpina* (Nygren, 1962), while in maize the B chromosomes are club shaped, missing any visible centromeric truncation (Randolph, 1941). In *S. purpureosericeum*, B chromosomes are indistinguishable from standard chromosomes, being of the same size and lacking any specific attribute. Sporadically, B chromosomes could be identified in meiotic metaphase I. Generally, they form ring bivalents when present in even numbers. Nevertheless, we observed that when a rod bivalent formation occurs, it is frequently composed of B chromosomes (Fig. 4B, C).

Unfortunately, all of these morphological features lose their significance and utility when it comes to the nuclei and observation of B chromosomes in interphase. To follow the supernumeraries through the cell cycle or at particular developmental stages of plants, more advanced markers must be employed. In animals, B-specific whole chromosome paints are very effective; in this way, the B chromosomes of the cichlid fish *Astatotilapia latifasciata* (Valente *et al.*, 2017) and of the characid fish *Astyanax scabripinnis* (Vicari *et al.*, 2011) were visualized. In plants, however, applying whole chromosome-specific probes is fraught with difficulty because of the extreme level of unspecific hybridization; hence, shorter probes specific for B chromosome segments are alternatively utilized in species, whereby the B chromosome sequence is acquired. To date, actually, this has been implemented in just a few species, namely rye (Martis *et al.*, 2012), maize (Alfenito and Bichler, 1993), goatgrass (Wu *et al.*, 2019), and meadow fescue (Ebrahimzadegan *et al.*, 2019)—in all of them, B-specific repeats were detected.

In our study, we broadened this not numerous group of species by adding to it *S. purpureosericeum*. The availability of B-specific PCR markers is crucial for further research of the *Sorghum* B chromosome, whether it is to facilitate the detection of B+ plants or to determine the number of B chromosomes within a plant, using either quantitative or digital droplet PCR. Here we provide reliable PCR-based markers with strong amplification. The set of primers was derived from nine putative B-specific clusters SpuCL115, SpuCL135, SpuCL144, SpuCL168, SpuCL169, SpuCL175, SpuCL189, SpuCL214, and SpuCL220 (Supplementary Tables S1, S2).

**Fig. 5.**
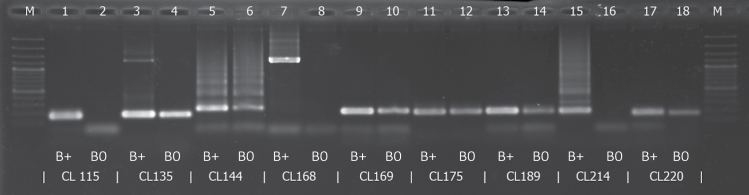
Electrophoresis of short amplicons for nine putative B-specific clusters (SpuCL115, SpuCL135, SpuCL144, SpuCL168, SpuCL169, SpuCL175, SpuCL189, SpuCL214, and SpuCL220). For all clusters, primer specificity was tested in parallel on B+ DNA and B0 DNA. The presence of PCR products of SpuCL115, SpuCL168, and SpuCL214 exclusively in B+ samples indicates their B specificity. The remaining primer pairs showed non-specific amplifications. The M lane corresponds to the GeneRuler 100 bp Plus DNA Ladder.

For the short amplicons, the amplification of only three primers (SpuCL115, SpuCL168, and SpuCL214) out of nine resulted in a product on the B+ template only. The others did not work specifically, in that they each showed amplification in both the B+ and B0 samples (Fig. 5).

**Fig. 6.**
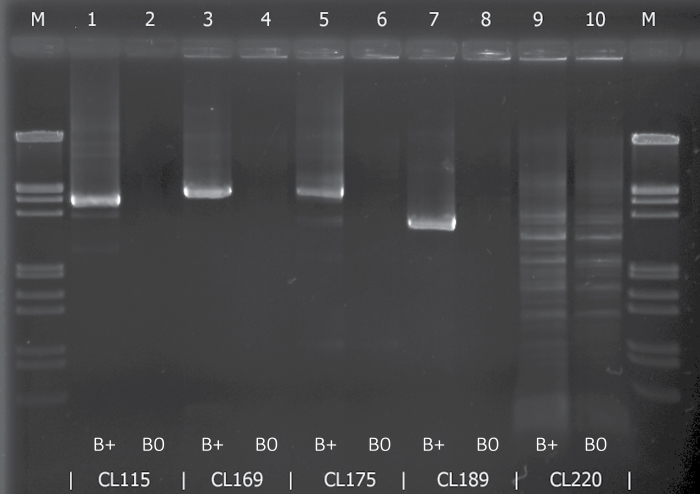
Electrophoresis of long amplicons for five putative B-specific clusters (SpuCL115, SpuCL169, SpuCL175, SpuCL189, and SpuCL220). Primer specificity was tested in parallel on B+ DNA and B0 DNA. The presence of PCR products of SpuCL115, SpuCL169, SpuCL175, and SpuCL189 exclusively in B+ samples indicates their B specificity. The M lane corresponds to λ DNA digested with *Eco*RI and *Hin*dIII.

Among the five primer pairs for the long amplicons, four were found to be B chromosome specific. Primers designed for clusters SpuCL115, SpuCL169, SpuCL175, and SpuCL189 provided products of an expected length from the B+ sample but no visible products for the B0 sample (Fig. 6). Primers derived from cluster SpuCL220 did not yield any B-specific products.

In aiming to track the B chromosome *in situ*, we developed strong and reliable cytogenetic markers specific for the B chromosome. Products of B-specific clusters SpuCL115, SpuCL168, SpuCL169, SpuCL175, SpuCL189, and SpuCL214 were labelled with Texas Red or FAM, and the probe specificity was tested on the meiocytes of a B+ plant. According to these results, SpuCL214 provided no distinct signal on any chromosome. Abundant signals for the Spu169, SpuCL175, and SpuCL189 clusters were detected on B chromosomes, but the probe also hybridized to chromosomes of the A complement. Only clusters SpuCL115 and SpuCL168 were confirmed to be B chromosome specific (Table 1). Hybridization of the DNA transposon SpuCL115 and satellite SpuCL168 showed a single chromosome-specific signal, which was regularly detected on one bivalent in the *S. purpureosericeum* plant with 2B chromosomes (Fig. 4A–C, G). Importantly, both clusters hybridized to the same chromosome (Fig. 4H). To address whether the probes targeted the B chromosome, in parallel we performed the identical experiment but using B0 plants, for which a signal was never found in their preparations (Fig. 4I). Both probes hybridized to the distal parts of opposite chromosome arms and represent a segment of significant size. However, the signal from cluster SpuCL115 was larger, more robust, and compact when compared with that of cluster SpuCL168. These results agree with the repeat analysis findings from before; specifically, they suggested that the representation of SpuCL115 amounted to 0.2% (4.2 Mbp in 1C) of the genome, being only 0.056% (1.2 Mbp in 1C) for cluster SpuCL168 (Table 1). Still, both signals were distinguishable on the nuclei as well (Fig. 4D, E, H). Nevertheless, the respective number of the loci varied due to the process of B elimination. When the micronucleus was present, the signal from the B-specific repeat was consistently detected here; when we followed the signals in mother nuclei and micronuclei, the sum of their signals was stable (Fig. 4E, F).

Our analysis revealed two specific FISH markers. While the signal of the satellite SpuCL168 is characterized by a rather dispersed structure, that of DNA transposom SpuCL115 is compact and ‘paints’ nearly a whole single arm of the B chromosome and so it is robust enough for use in various applications. Similar blocks of repeats were found in *Festuca pratensis*, whereby, when using two satellite probes making up 0.33% and 0.015% of its genome, respectively, practically the whole B chromosome can be visualized (Ebrahimzadegan *et al.*, 2019). In contrast, no B-specific block of similar size was found, for example, in *Ae. speltoides*, where two B-specific satellites represented only 0.053% and 0.026% of the genome (Wu *et al.*, 2019) whose signals had a less dense, ‘dotty’ pattern.

Additionally, cluster SpuCL4 proved to be a centromeric probe, able to hybridize to all centromeres. Nonetheless, its signal is not uniform. There are two chromosome pairs with a unique hybridization pattern: the signal of the B centromere is significantly larger, and the difference is so unambiguous that it could also serve as a marker for B chromosome identification (Fig. 4A–C). Next to the B chromosome, one chromosome pair from the A complement showed a remarkably weaker and smaller signal relative to the remaining bivalents.

### An adverse effect of the B chromosomes on a host

**Fig. 7.**
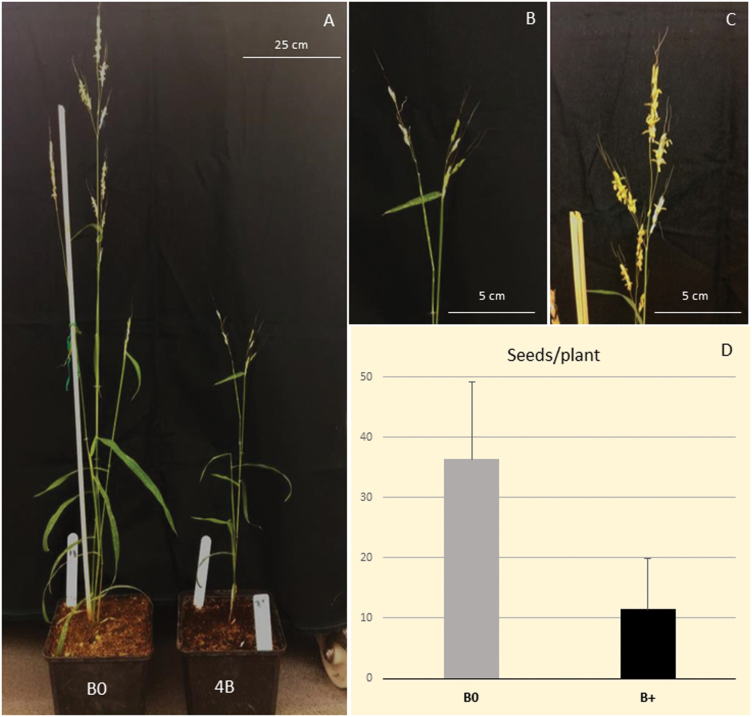
Harmful effects of the B chromosome presence on phenotype. A 4B plant whose fitness was significantly decreased when compared with a B0 plant (A); spikes of a 4B plant characterized by morbid development and reduced fertility (B); normal inflorescence structure of a B0 plant (C); and comparison of seed production of 11 B0 and 2B plants (D).

The harm of a high dosage of B chromosomes on the fitness of their carriers is generally known, for which the onset of a negative effect on phenotype is anticipated just in higher dosage (Valente *et al.*, 2017). A maximum of 6Bs were observed in *S. purpureosericeum* (Janaki-Ammal, 1940). Nonetheless, the phenotypic effect of B chromosomes in *S. purpureosericeum* was apparent even under a disomic constitution, when plants with 2Bs were mostly smaller and their fertility was decreased, and the effect was even stronger in plants possessing 3Bs or 4Bs (Fig. 7A–C). Plants with 2Bs produced a significantly lower number of seeds when compared with B0 individuals (Fig. 7D; Supplementary Table S3). The seed production was evaluated for 11 B+ and 11 B0 plants. While B0 plants produced 36.4±12.8 seeds, B+ plants have significantly (Student’s t-test, *P*<0.001) reduced fertility, producing only 11.5±8.4 seeds. In rye, wherein a maximum of 8Bs was reported, harbouring 3Bs in its genome is not harmful and the phenotype is identical to that of the B0 plant (Jones *et al.*, 2008). Nonetheless, our findings are in line with earlier work by Magoon *et al.* (1961), who reported a >95% sterile tiller in *S. purpureosericeum* plants having 3Bs.

We assumed that the onset of the adverse effects of B chromosomes on host plant vigour depends on the proportion of their DNA in the nucleus rather than the number of B chromosomes *per se*. According to earlier research on rye and maize, reductions in plant fitness are pronounced when the B chromosome mass exceeds 20% of DNA in the nucleus, a potential heuristic obtainable via calculations of previously published data. In maize with a genome size of 4.8 Gbp/2C (Schnable *et al.*, 2009) and a B chromosome size estimated at 140 Mbp (N. Blavet *et al*., unpublished results), poor fitness and reduced fertility were observed in plants with 10 or more B chromosomes (Masonbrink et Birchler, 2012). In the 10Bs plant, B chromosomes constituted ~23% of DNA in the diploid nucleus. Similarly, in rye (15.8 Gbp/2C; Bartos *et al*., 2008), where B chromosome size was reported to be 540 Mbp (Martis *et al.*, 2012), the presence of 6Bs (~20% of diploid DNA content) was shown to be detrimental (Jones *et al.*, 2008). We also observed a dramatic impact on plant fertility and condition when the genome carried two or more B chromosomes. We estimated the genome size of *S. purpureosericeum* (accession no. IS18947) to be 4.42 Gb/2C (Supplementary Fig. S3; Supplementary Table S4), which is similar to previous estimates (4.2 Gbp/2C; Price *et al.*, 2005). Together with B chromosome size in this species being 421 Mbp (Supplementary Fig. S3; Supplementary Table S5), the accrued mass of 2Bs accounted for ~20% of extra DNA in the diploid nucleus. In conclusion then, the proportion of DNA content of 2Bs in relation to the genome size of *S. purpureosericeum* is comparable with that of 10Bs in maize or 6Bs in rye plants, and, as such, the substantial fitness impact of B chromosomes in *S. purpureosericeum* is not all that surprising.

Counterintuitively, this feature (i.e. the large size of *S. purpureosericeum* B chromosome) runs against all mechanisms the B chromosomes have evolved to themselves in hosting individuals. Any degree of sterility adversely affects the transmission of B chromosomes into the progeny and lessens their probability of being retained in the population. Taking this finding into due consideration, the reduction of B chromosome size would seem to be a meaningful evolutionary trend.

## Supplementary data

The following supplementary data are available at *JXB* online.

Fig. S1. Detection of B+ nuclei in specific parts of a 2B plant using flow cytometry.

Fig. S2. Topology of the putative B-specific clusters

Fig. S3. Determination of genome size of a B0 plant of *Sorghum purpureosericeum* and the size of the B chromosome.

Table S1. List of primers designed for the short amplicons.

Table S2. List of primers designed for the long amplicons.

Table S3. Seed production of B0 and 2B plants.

Table S4. Genome size of B0 plants of *Sorghum purpureosericeum*

Table S5. Size of the B chromosome of *Sorghum purpureosericeum.*

eraa548_suppl_Supplementary-Figures-S1-S3_and_Tables-S1-S5

## Data Availability

Sequence reads used in this work, the resulting sequences of clusters, and the counts of B+/B0 reads in each cluster have been submitted to the Dryad Digital Repository (https://doi.org/10.5061/dryad.rxwdbrv5j; Karafiátová et al., 2021).
