# Supplementary material for: The B chromosome of Sorghum purpureosericeum reveals the first pieces of its sequence
Source: J Exp Bot. 2020 Nov 20;72(5):1606–16. doi: 10.1093/jxb/eraa548 (PMC7921303; doi:10.1093/jxb/eraa548)
Supplement: eraa548_suppl_Supplementary-Figures-S1-S3_and_Tables-S1-S5 [file eraa548_suppl_supplementary-figures-s1-s3_and_tables-s1-s5.pdf]

Fig. S1. Detection of B+ nuclei in specific parts of 2B plant using flow-cytometry. Histograms represent the results of analysis of the nuclei in the samples prepared from the following tissues: root (A), stem (B), leaf (C), leaf meristem (D), last node (E) and peduncle (F). In histograms D-F, the population of B+ nuclei is pointed by the arrow. The main peak in all histograms corresponds to B0 nuclei.

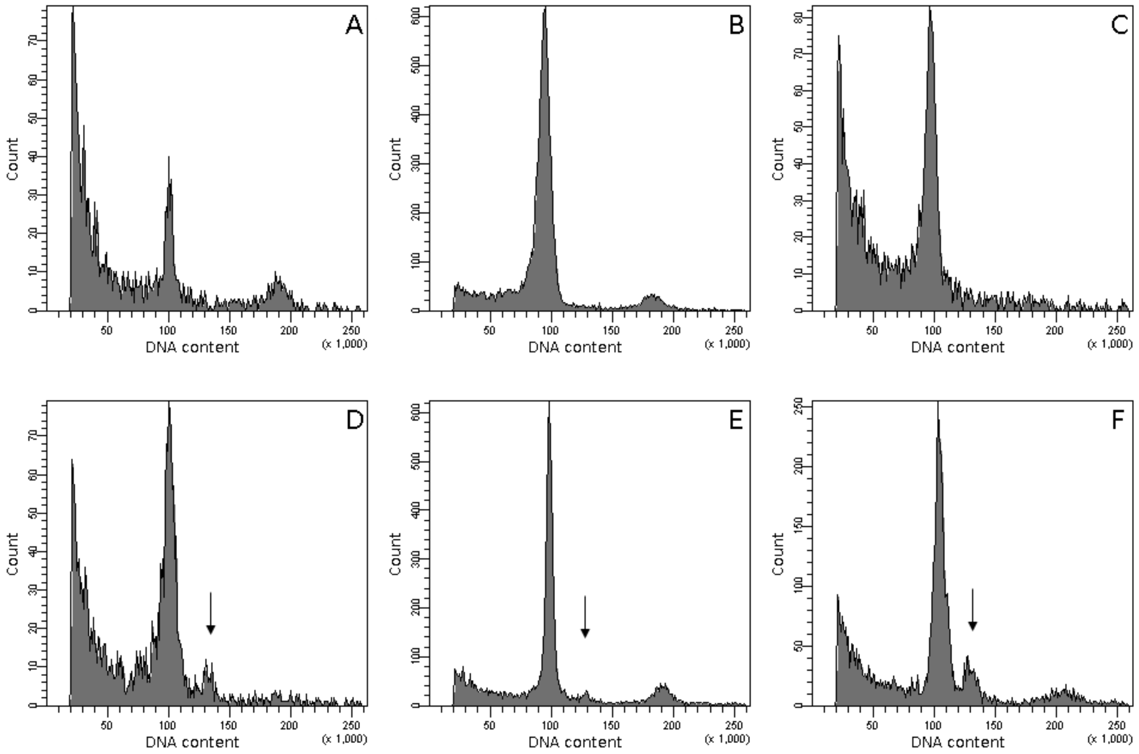

Fig. S2. Topology of the putative B-specific clusters. Overlapping reads were clustered using graph-based approach in RepeatExplorer2 pipeline. Circular graphs indicate tandem repeat organization for clusters SpuCL135, SpuCL144, SpuCL168, SpuCL169, SpuCL189 and SpuCL214.

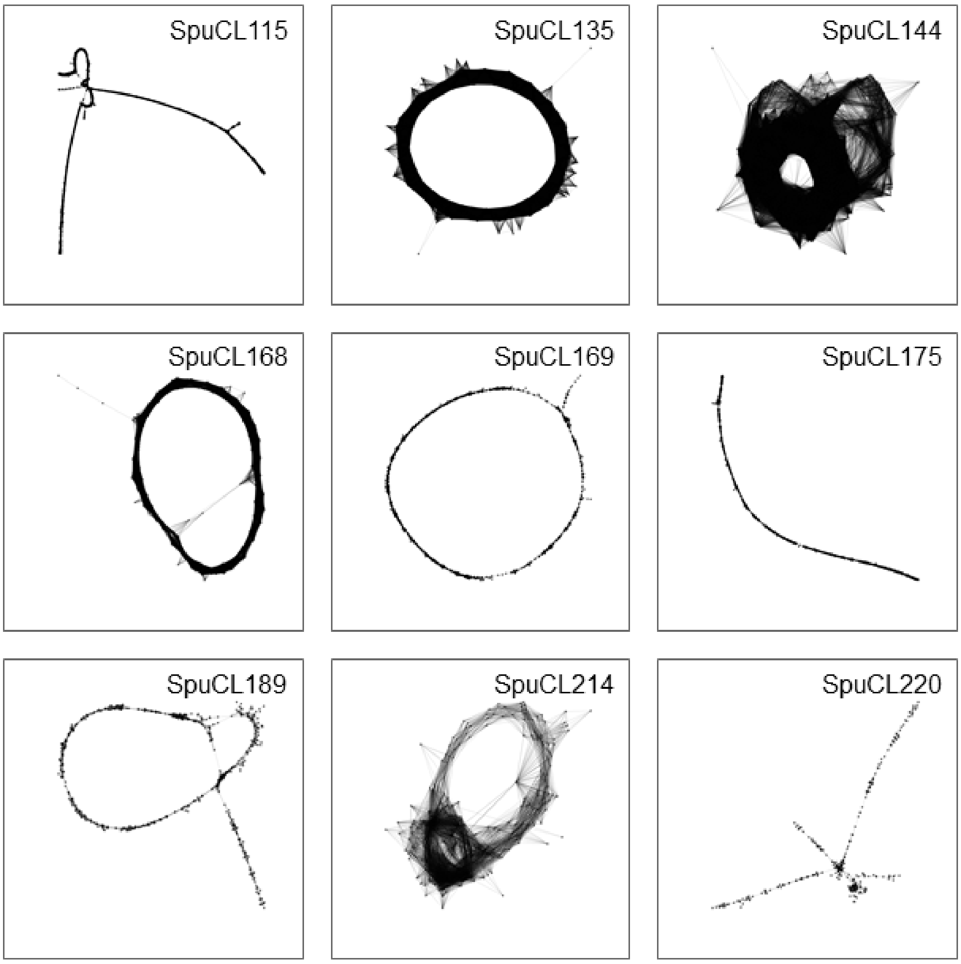

Fig. S3. Determination of genome size of B0 plant of *Sorghum purpureosericeum* and the size of the B chromosome. (A) For genome size estimation nuclei were simultaneosly isolated from leaves of *S. purpureosericeum* (without B chromosome) and *Zea mays* cv. CE-777 and stained with propidium iodide according to Doležel *et al.* (2007). First and second peak in histogram represent population of 2C nuclei of *S. purpureosericeum* and *Zea mays*, respectively. (B) For estimation of B chromosome size, identical protocol was used, but nuclei were isolated from florets of *S. purpureosericeum* plant possesing one B chromosome. First and second peak in histogram represent population of 2C and 2C+1B nuclei of *S. purpureosericeum*, respectively.

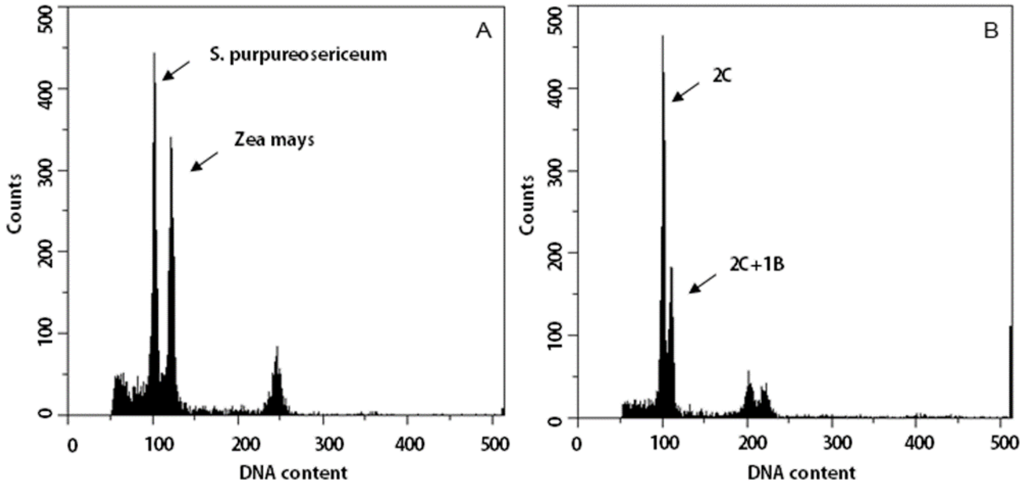

Table S1. List of primers designed for the short amplicons

Cluster #	Name of primer	Primer sequence 5´-->3´	Amplicon length (bp)
SpuCL115	CL115ctg8_2F<br>CL115ctg8_2R	GCCAGGTGCGTAATTGTTTT<br>AAGTTTTGCAACGAGCCACT	154
SpuCL135	CL135_2F<br>CL135_2R	GGCAACTGGACGAGAATAGG<br>TCACGCTGTAGGTGTTGAGG	174
SpuCL144	CL144_2F<br>CL144_2R	ATTGCTGACTCGACCCATTT<br>TGCTAGGCCCCTATAGAAACTG	230
SpuCL168	CL168_2F<br>CL168_2R	CGCTGCTGCTTGTTTTGATA<br>GGGTGTTCAAAGGTGATGCT	1290
SpuCL169	CL169_2F<br>CL169_2R	TGTAAGCTGTGCAGGATTGG<br>CATGCCAGTGTTGAACCATC	199
SpuCL175	CL175_2F<br>CL175_2R	TCATCTTCTCCGGTGAATCC<br>ACCACCTCTCTGCTGCATCT	189
SpuCL189	CL189_2F<br>CL189_2R	ATGCCCATAACCTGAGATGC<br>GTGGTTGCTCTGGGACAAAT	186
SpuCL214	CL214_2F<br>CL214_2R	GCCACATGCTTCATTTCCTT<br>GAGCACATGCCGGTGTAAT	193
SpuCL220	CL220ctg4_2F<br>CL220ctg4_2R	GACGGGAGGTCAGTTTGGTA<br>TTCCAGCTCCCACACTCTCT	156

Table S2. List of primers designed for the long amplicons

Cluster #	Name of primer	Primer sequence 5´-->3´	Amplicon length (bp)
SpuCL115	CL115_1F<br>CL115_1R	TCGCTCTCGTCATGTTTGTC<br>CTTGTCCGATGCAATTTGTC	4100
SpuCL169	CL169_1F<br>CL169_1R	ATGGTTCAACACTGGCATGA<br>TGTGCCTTGTTTGAGTCAGC	4454
SpuCL175	CL175_1F<br>CL175_1R	GCTCCACGCTTGGTCTCTAC<br>TGCCACCAGAGAAGTGTGAG	4578
SpuCL189	CL189_1F<br>CL189_1R	TATTGAGGCTTTGGCACCTT<br>TTTAGGTCCCTTTGCCCTTT	3095
SpuCL220	CL220ctg4_1F<br>CL220ctg4_1R	AGAGAGTGTGGGAGCTGGAA<br>TGAACGCTTAAGCACGAATG	2368

Table S3. Seed production of B0 and 2B plants

B0 plants		2B plants	
plant No.	seeds	plant No.	seeds
1	18	1	6
2	53	2	20
3	35	3	3
4	48	4	3
5	15	5	24
6	23	6	14
7	31	7	3
8	52	8	1
9	38	9	11
10	48	10	17
11	39	11	24

Table S4. Genome size of B0 plant of *Sorghum purpureosericeum*

Plant No.	sample [peak]	standard [peak]	peak ratio	**2C DNA [Gb]**
1	104.93	125.21	0.8380	4.45
1	104.37	125.81	0.8296	4.41
1	100.91	122.23	0.8256	4.38
2	102.36	121.93	0.8395	4.46
2	101.70	121.87	0.8345	4.43
2	105.98	127.04	0.8342	4.43
3	97.47	116.88	0.8339	4.43
3	98.48	119.74	0.8224	4.37
3	99.75	120.71	0.8264	4.39
**mean**				**4.42**
**±SD**				0.03

Genome size was calculated based on position of peaks of *S. purpureoseticeum* B0 plant (sample) and *Zea mays* cv. CE-777 (standard) in the histogram obtained using flow cytometry (see Fig. S3 for more details). The genome size of *Zea mays* cv. CE-777 (5.43 pg/2C) was determined previously (Lysák and Doležel, 1998). The calculation was done considering 1 pg DNA is equal to 0.978×10^9 bp (Doležel *et al.*, 2003).

Table S5. Size of the B chromosome of *Sorghum purpureosericeum*

Plant No.	sample [peak]	standard [peak]	peak ratio	2C DNA [Gb]	**B chr size [Mb]**
1	111.31	101.62	1.0954	4.84	421
1	110.51	101.09	1.0932	4.83	412
1	111.44	101.59	1.0970	4.84	428
2	110.49	100.43	1.1002	4.86	442
2	112.73	103.01	1.0944	4.83	417
2	111.12	101.64	1.0933	4.83	412
3	111.30	101.67	1.0947	4.83	418
3	111.75	101.85	1.0972	4.85	429
3	110.48	101.05	1.0933	4.83	412
**mean**					**421**
**±SD**					10

B chromosome size was calculated based on position of peaks of *S. purpureoseticeum* B+ (sample) and B0 (standard) nuclei in the histogram obtained using flow cytometry (see Fig. S3 for more details). For the calculation, we determined genome size of *S. purpureoseticeum* B0 plant to be 4.42 Gb/2C (see Table S4).

Doležel J, Bartoš J, Voglmayr H, Greilhuber J. 2003. Nuclear DNA content and genome size of trout and human. Cytometry Part A.51A, 127–128

Doležel J, Greilhuber J, Suda J. 2007. Estimation of nuclear DNA content in plants using flow cytometry. Nature Protocols 2, 2233–2244

Lysák MA, Doležel J. 1998. Estimation of nuclear DNA content in Sesleria (Poaceae). Caryologia 52: 123 - 132 (1998)
